# Exploring Patient and Caregiver Perceptions of the Facilitators and Barriers to Patient Engagement in Research: Participatory Qualitative Study

**DOI:** 10.2196/79538

**Published:** 2025-09-30

**Authors:** Sasha Melanda Kullman, Louise Bird, Amy Clark, Amanda Doherty-Kirby, Diana Ermel, Nathalie Kinnard, Marion Knutson, Andrew Milroy, Lesley Singer, Anna Maria Chudyk

**Affiliations:** 1 College of Pharmacy, Rady Faculty of Health Sciences, University of Manitoba, Apotex Centre, 750 McDermot Avenue West, Winnipeg, R3E 0T5, Canada, 1 204-474-9306; 2 Patient-Caregiver Partner, College of Pharmacy, Rady Faculty of Health Sciences, University of Manitoba, Winnipeg, MB, Canada

**Keywords:** participatory research, patient-oriented research, public and patient engagement, patient partnership, co-analysis

## Abstract

**Background:**

Patient engagement in research is the meaningful and active involvement of patient and caregiver partners (ie, patients and their family or friends) in research priority-setting, conduct, and governance. With the proper support, patient and caregiver partners can inform every stage of the research cycle, but common barriers often prevent their full engagement.

**Objective:**

This participatory qualitative study aimed to answer the question: What are the facilitators and barriers to patient engagement experienced by patient and caregiver partners in a Canadian research context?

**Methods:**

Participants were N=13 patient and caregiver partners (median age 62 y, IQR 58-69 y; 11/13, 85% women; 13/13, 100% White) from 4 provinces who completed 60‐90-minute semistructured videoconferencing interviews. The interviews were transcribed verbatim. A researcher and a patient partner reviewed the transcripts and curated a dataset of 90 participant quotations representing facilitators and barriers to patient engagement. This dataset was co-analyzed using participatory theme elicitation alongside 7 patient and caregiver partners with diverse identities who were not among the participants we interviewed and, therefore, contributed novel perspectives.

**Results:**

We generated four themes depicting factors that facilitate meaningful patient engagement alongside barriers that arise when these factors are not in place: (1) Co-defining roles and expectations; (2) demonstrating the value and impact of engagement; (3) psychological safety; and (4) community outreach, training, and education. We then discuss how barriers to enacting these 4 factors can be mitigated and provide a practical checklist of considerations for both researchers and patient and caregiver partners for engaging together throughout the research cycle.

**Conclusions:**

Research teams conducting patient and caregiver engagement activities should draw from our findings to mitigate barriers and facilitate meaningful engagement experiences.

## Introduction

Patients and their care partners (family or friends) offer unique insights into the health care system, making them key contributors to health research. By collaborating with researchers, patient and caregiver partners ensure that research addresses the needs and priorities of patients and their families, ultimately improving the health care system. In Canada, this is known as patient engagement—the meaningful and active engagement of patients and their care partners as co-researchers throughout the research cycle, ideally beginning at the grant-writing stage [1]. This concept may be referred to as patient and public involvement [2] or consumer involvement [3].

Individuals who share their lived or living experiences to inform a health research project may be given various titles depending on their role or identity, such as patient, caregiver, family member, or person with lived experience. These titles may also reflect their level of involvement, including terms like collaborator or partner. In this study, the terms “patient partner” and “caregiver partner” refer to individuals with experience navigating health or the health care system, either directly or as a caregiver.

Engaging patient and caregiver partners can occur at any stage of and throughout the research cycle, from planning to knowledge mobilization [1]. The benefits of patient engagement range from improving recruitment [4] to personal gains for researchers (eg, increased confidence) and patient or caregiver partners (eg, community building) [5]. Given these advantages, major research funders now often require patient and caregiver engagement in funding calls [1]. However, some researchers still face challenges with engagement, such as a lack of time, funding, training, or institutional support [6-8]. These barriers can lead to tokenistic involvement and dissatisfaction among patient and caregiver partners [9].

To improve their practice, researchers can learn from studies on the barriers and facilitators to patient engagement, including perspectives from researchers [8], patient and caregiver partners [9], and combined viewpoints [10]. However, only a few studies have captured the perspectives of patient and caregiver partners by involving them on the research team [5,7,9], and more co-led studies on patient and caregiver engagement are needed. In addition, most studies are from the United Kingdom, with fewer focusing on the Canadian context. As engagement practices vary by country, more research in Canada is needed.

This qualitative study, which was co-designed, co-led, and co-authored by patient and caregiver partners, examines the barriers and facilitators to patient engagement in Canada. Our primary research question is: What are the key barriers and facilitators to patient engagement experienced by patient and caregiver partners, and how do these impact their perceptions of engagement?

## Methods

### Research Team

This study was led by AMC, an early career patient-oriented researcher with expertise in patient engagement, and SMK, a PhD student who was the patient engagement liaison and oversaw all engagement activities. Both researchers identified as women. A total of 8 individuals with previous experience of research partnering (ie, LB, AC, AD-K, DE, NK, MK, AM, and LS) were engaged throughout the study. We refer to this group as patient-caregiver partners, but they identified with a variety of terms to describe their research involvement, as shown in Multimedia Appendix 1. We specifically chose the term “patient-caregiver” because the individuals we partnered with had experience as both patients and caregivers. Within their range of experience, 4 of our research partners primarily identified themselves as patient partners, while the other 4 identified equally with the patient and caregiver partner role.

Collectively, the 8 patient-caregiver partners had between 1.5 and 32 years of experience in research partnering and resided across 5 provinces in Eastern and Central Canada. A total of 4 patient-caregiver partners lived in urban areas and 4 lived in semiurban or rural areas. A total of 6 patient-caregiver partners identified as women, 1 as a transgender woman, and 1 as a man. Collectively, the patient-caregiver partners had a wealth of experience in research partnering across health care domains, including basic science, cancer, chronic pain, pediatric disability, mental health, patient safety, health care provider education, and health care policy. Their contributions included informing clinical trials, knowledge synthesis and mobilization, reviewing research grants, co-developing educational modules, co-chairing committees, and shaping national and provincial health initiatives. The patient-caregiver partners’ past research experiences, diverse backgrounds, linguistic perspectives, and direct experiences with health care informed our research findings.

### Philosophical Approach

This research is informed by critical realism, a philosophical approach that acknowledges an independent reality while emphasizing the need to explore underlying mechanisms that shape observable experiences [11]. Within this critical realist approach, our stratified realist ontology supports the idea that engaging patient and caregiver partners in research helps bring researchers closer to understanding the truth of their experiences. Our constructivist epistemology also acknowledges that experiences vary between individuals [11]. Therefore, incorporating diverse patient and caregiver partner perspectives through interviews and participatory analysis should help us capture the complex factors that shape their experiences.

### Participants and Data Collection

This research is part of a larger 3-part project exploring the current and preferred future states of patient engagement in research in Canada [12-14]. Participants (N=13) were patient and caregiver partners with previous experience engaging in research funded by the Canadian Institutes of Health Research through the Strategy for Patient Oriented-Research [1] who completed a cross-sectional survey assessing the activities and impacts of patient engagement [12]. These individuals subsequently agreed to participate in a qualitative interview about their engagement experiences [13]. Details of participant recruitment are described elsewhere [13]. After providing written informed consent, all participants completed a 60‐ to 90-minute semistructured interview via videoconferencing. Each interview was co-facilitated in English by an academic researcher (AMC) and a patient partner (Mr. Roger Stoddard). Interviews were audio-recorded, transcribed verbatim, and all identifying information was removed from the transcripts to protect participants’ privacy. Participants member-checked summaries of the interview data using a participatory process in which they refined the data and informed future directions for the analysis [15].

### Ethical Considerations

All members of the research team (including patient-caregiver partners) completed research ethics training, privacy training, and signed oaths of confidentiality before engaging in research activities. The data was deidentified to protect participants’ privacy, and their names were replaced with confidential participant numbers. Both patient-caregiver partners and research participants provided written informed consent to ensure they understood and were comfortable with their respective roles in the study. The University of Manitoba Health Research Ethics Board approved our work with patient-caregiver partners (Protocol HS26450; H2024:142). In addition, the University of Manitoba Human Ethics Board approved the study from which we collected our qualitative data (Protocol E2019:082; HS23180). All patient-caregiver partners were compensated at a rate of CAD $25 (approximately US $18) per hour. Research participants were compensated with CAD $75 (approximately US $54) in total.

### Patient Engagement in This Study

**Figure 1.**
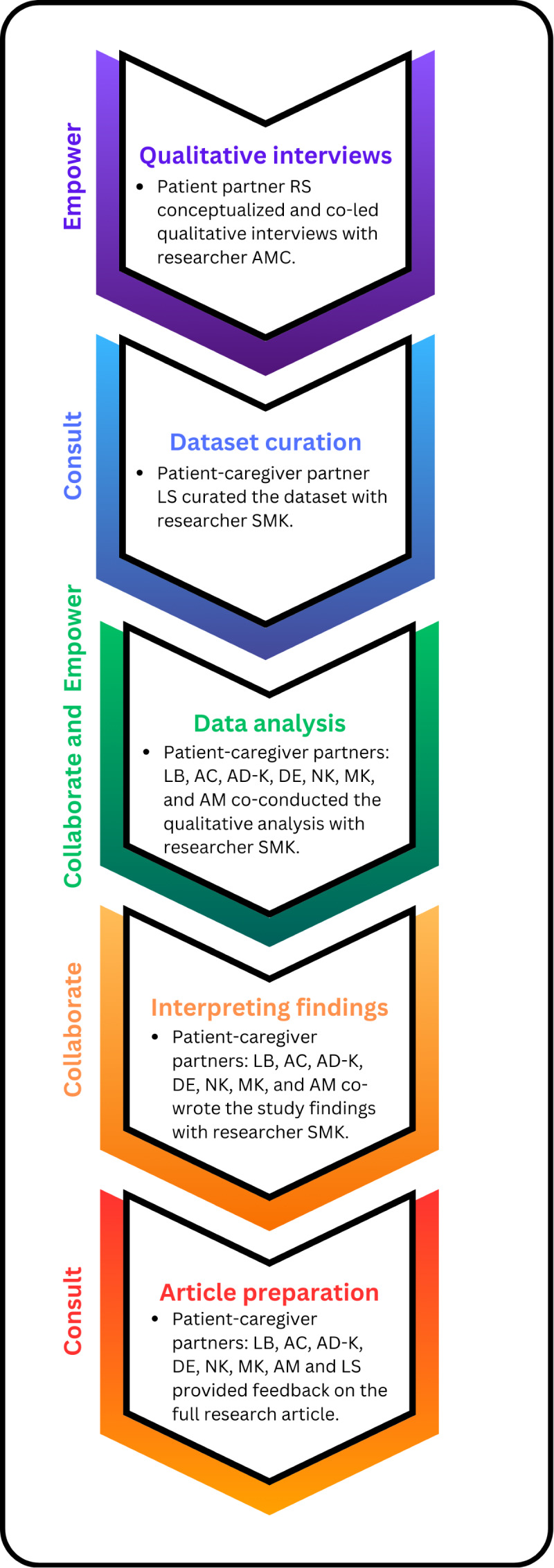
Patient-caregiver partner engagement throughout the research process.

This research was conceptualized and driven by patient and caregiver partner collaboration. The larger qualitative study from which we drew our data was initiated by a patient partner (Mr. Roger Stoddard) in collaboration with the senior author (AMC). Examining barriers and facilitators to patient engagement was identified as a priority by patient and caregiver partner participants who member-checked the qualitative data [15]. In this study, 8 patient-caregiver partners were engaged at the levels of “consult,” “collaborate,” and “empower” [16] throughout each stage of the research process, as described in the following sections and Figure 1. We report our patient engagement activities using the Guidance for Reporting Involvement of Patients and the Public, Version 2 (GRIPP2) checklist [17] (see Checklist 1) and qualitative research process using the Consolidated criteria for Reporting Qualitative research (COREQ) checklist [18] (see Checklist 2).

### Patient-Caregiver Partner Recruitment

Patient-caregiver partners were recruited through social media and email newsletter advertisements. An additional patient-caregiver partner (LS), who had participated in the larger qualitative study, was directly recruited to assist with dataset curation (in addition to article preparation) due to her familiarity with the interview data. Before the project began, SMK engaged all patient-caregiver partners in an introductory and icebreaker meeting where they co-created a “Terms of Reference” document (also known as a “team charter”) outlining the project’s aims, proposed team member roles, and how the group would foster mutual respect, co-building, support, and inclusion [19]. Specific actions we took to promote these values included having 5‐10 minute informal check-ins at the start of each meeting, accommodating all schedules by offering 2 options for each meeting (1 on a weekday and 1 on a weekend), sharing written materials at least 5 days before meetings, sending frequent reminders, discussing compensation and reimbursement openly, and setting guidelines for respectful communication. All patient-caregiver partners waived the option to remain anonymous so they could be recognized as co-authors.

### Analysis: Participatory Theme Elicitation

In collaboration with our patient-caregiver partners, we analyzed data from the larger qualitative study using participatory theme elicitation (PTE) [20]. The 5 steps of PTE are described below:

#### PTE Step 1: Dataset Selection

In PTE step 1, a subset of data from the 13 interview transcripts was curated for analysis. SMK consulted with patient-caregiver partner LS across three 2-hour videoconferencing meetings to complete this step. Quotations were excluded from the analysis if they could not be understood as standalone statements, lacked relevance to the research question, were repetitive, or came from frequently quoted participants when less vocal participants expressed similar ideas. In total, n=94 quotations were selected for analysis. These quotations were then reviewed by the 7 patient-caregiver partners (LB, AC, AD-K, DE, NK, MK, and AM) who would collaborate on the data analysis. These patient-caregiver partners removed 4 quotations from the dataset due to their lack of relevance or clarity and expanded 17 quotations to include more context. A final sample of n=90 quotations was subjected to the PTE analysis.

#### PTE Steps 2 and 3: Capacity Building and Open Sorting

In PTE step 2, SMK trained the patient-caregiver partners to perform step 3 of PTE, open sorting, using a practice data sorting activity. In PTE step 3, the 7 patient-caregiver partners and SMK independently sorted the quotations in the dataset into groups they found conceptually similar, using any criteria they found meaningful [20]. All data sorting was conducted using Miro whiteboards, a web-based collaboration platform used in past PTE research [21]. Each team member completed this step over a 4-hour period following the instructions in Multimedia Appendix 2.

#### PTE Step 4: Data Grouping

In PTE step 4, all group members’ individual open sorting decisions were recorded in a spreadsheet (see Multimedia Appendix 3 ). A network analysis was used to generate a consensus of the independent card-sorting decisions made during PTE step 3 [20]. The analysis produced 4 clusters of quotations, representing candidate themes that emerged from the sorting process. These clusters were visualized using the network diagram shown in Multimedia Appendix 4. In this diagram, each quotation was represented by a colored node. The node color, spatial distance between nodes, and the lines connecting them indicated how frequently different quotations were sorted together by patient-caregiver partners. This step helped ensure all research team members contributed equally to initial theme generation [20].

#### PTE Step 5: Data Analysis and Interpretation

In PTE step 5, SMK and the 7 patient-caregiver partners collaborated over six weekly 2-hour meetings to refine the candidate themes from PTE step 4, creating a final set of themes that addressed the research question [20]. To support this process and group discussion, a Miro Whiteboard was created containing all 90 quotations, color-coded according to the network diagram’s clusters (see Multimedia Appendix 5). Each meeting followed a structured approach: SMK began by reading aloud all quotations within a cluster and prompting patient-caregiver partners to share their initial impressions. Then, using the “tag” function in Miro, patient-caregiver partners collaboratively tagged each quotation with descriptive words to highlight recurring ideas that could inform themes or subthemes, allowing them to build on each other’s insights. During this process, patient-caregiver partners could also move quotations from their original cluster to a different cluster or create new clusters if needed. Once all quotations were tagged and patient-caregiver partners were satisfied with the quotation groupings, they named each theme and subtheme.

## Results

### Overview of Results

The qualitative dataset used in the present analysis contained data from N=13 participants who identified as patient (9/13; 69%) or caregiver (4/13; 31%) partners. Participants had a median age of 62 years (IQR 58-69 y), 11/13 (85%) identified as female, and 2/13 (15%) identified as male. All participants identified as White, and a majority had completed a master’s degree as their highest level of education (8/13, 62%). Participants were located in 4 Canadian provinces: Ontario (7/13, 54%), Alberta (3/13, 23%), British Columbia (2/13, 15%), and Québec (1/13, 7%). The collaboration with patient-caregiver partners who co-conducted this research added additional perspectives from the provinces of Saskatchewan, Manitoba, Ontario, Québec, and Prince Edward Island.

**Figure 2.**
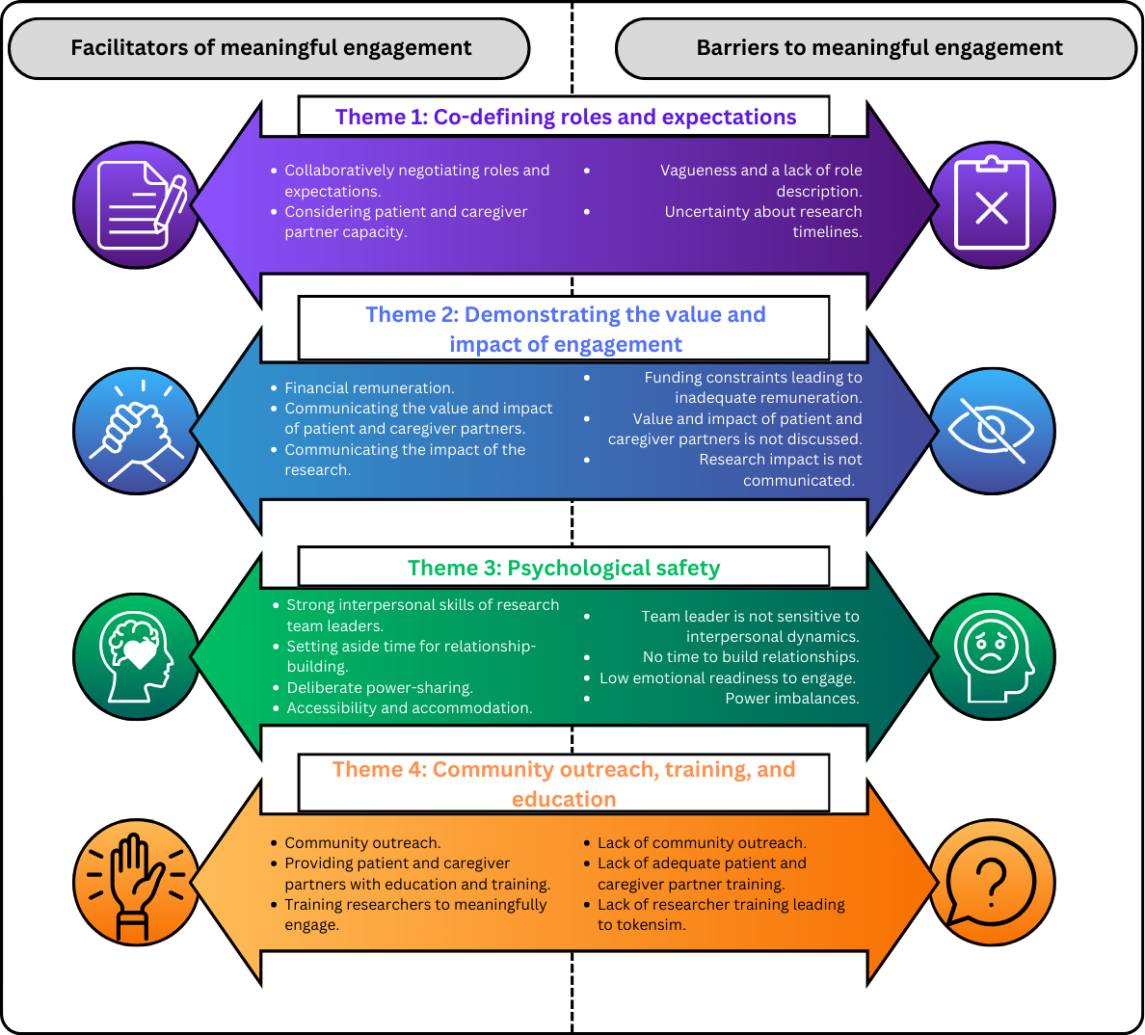
Summary of thematic results.

In collaboration with patient-caregiver partners, 4 themes were generated depicting factors that facilitate meaningful patient engagement alongside barriers that arise when these factors are not in place: (1) Co-defining roles and expectations; (2) demonstrating the value and impact of engagement; (3) psychological safety; and (4) community outreach, training, and education (see Figure 2). Within these themes, 14 subthemes are discussed.

### Theme 1: Co-Defining Roles and Expectations

The first theme highlights how patient engagement is facilitated when roles and expectations are established collaboratively. Within this theme, 3 subthemes are explored: (A) the necessity of co-defining roles and expectations, (B) the value of discussing the capacity of patient and caregiver partners, and (C) instances where roles and expectations were not clearly defined.

#### Subtheme A: “It Has to Be a Discussion and a Negotiation”—The Need to Co-Define Roles and Expectations

Defining the role of patient and caregiver partners should involve a collaborative negotiation. By working together with researchers to clarify roles and expectations at the research outset, patient and caregiver partners can better contribute their lived or living experiences and professional expertise (if applicable) to the research process. As Participant 4 described, this approach ensures that the skills and interests of a patient and caregiver partners align well with the research activities. It also provides them with the opportunity to decline roles that are not a good fit:

I think [patient engagement] has to be a deliberate matching of the skills, interests, and aptitudes of that [patient or caregiver] partner with the purpose that you’re drawing them in at the particular stage of research.

[Participant 4]

Flexibility in defining roles is also key. Researchers should have a vision for their engagement but remain open to patient and caregiver partners’ input. Communication and trust are fostered when patient and caregiver partners can collaboratively negotiate their roles. To achieve clear role expectations, participants recommended co-developing a “team charter” or “terms of reference”—a document outlining the goals, scope, and expectations within a research project. This process can help patient and caregiver partners understand where they fit within the research and encourages researchers to explore new ways of engaging with them. It can also be helpful to appoint one research team member as the “patient engagement liaison” who oversees all engagement activities and addresses questions or concerns from patient and caregiver partners.

#### Subtheme B: “You Want to Do Your Part, but It Can Take Its Toll”—Considering Patient and Caregiver Partner Capacity

Patient and caregiver partners often balance research engagement with employment, personal health responsibilities, caregiving duties, or simultaneous engagement in other studies. When defining roles, it is crucial to work with patient and caregiver partners and consider the extent they wish to be engaged in a study. For example, some may hesitate to decline opportunities due to their passion for research and advocacy, even when they are at capacity. Relatedly, less experienced patient and caregiver partners may worry that declining one research opportunity will result in fewer offers in the future, leading to situations of overwork:

Being able to say “no” to doing more and more is a challenge. Because you want to help, and you want to push the science, and the collaboration between patients and researchers and clinicians working together more in the future. You want to do your part, but it can take its toll also in terms of pain and fatigue.

[Participant 5]

On the opposite end of the spectrum, some patient and caregiver partners may want to expand their role but are not given opportunities to do so because researchers fear overburdening them or underestimate their capacity to take on more research tasks. Researchers can support patient and caregiver partners by providing clear information about time commitments, responsibilities, and opportunities to get involved, ensuring they can tailor their role to suit their capacities and interests. Since capacity can change over time, all members of the research team should be open to rediscussing and adjusting their roles as needed.

#### Subtheme C: “Vagueness and Lack of Role Description”—When Roles and Expectations Are Not Clearly Defined

When roles and expectations are not established collaboratively, barriers to engagement may arise. As noted by Participant 13, a lack of clear, co-defined roles can make patient and caregiver partners feel like tokenistic members of the research team who are kept at arm’s length from the project.

We [were] given the task, which was very vague, ‘tell us how patients should be involved?’ But it seemed like none of our suggestions were going anywhere… We started with about half a dozen [patient and caregiver partners] and we’re down to two… I assume it had something to do with the vagueness and the lack of a role description.

[Participant 13]

Uncertainty around research timelines also creates engagement challenges. Patient and caregiver partners may not anticipate how long research projects take due to ethics reviews, publication processes, and other delays. Regular timeline updates and ongoing education about research stages help patient and caregiver partners stay informed. When these updates are absent, partners may feel disconnected from their roles or struggle to balance their other commitments.

### Theme 2: Demonstrating the Value and Impact of Engagement

The second theme highlights how understanding the value and impact of one’s role enhances patient engagement. Within this theme, we discuss 2 subthemes: (A) monetary compensation and (B) communicating the impact of patient and caregiver partners as well as the broader impact of the research. The third subtheme explores (C) how a lack of clarity regarding the value of their role can negatively affect patient and caregiver partners.

#### Subtheme A: “Patient Expertise Is Expertise, and It Should Be Compensated and Acknowledged in Its Own Right”—The Importance of Financial Remuneration

Patient and caregiver partners often join research projects with an altruistic desire to improve health research. However, altruism does not equate to volunteerism, and compensating patient and caregiver partners through financial remuneration or other ways of showing appreciation (eg, donations, training opportunities) is an important facilitator of engagement. Compensation validates partners’ lived or living expertise and acknowledges the greater mental and emotional burden they may carry as team members who share their experiences to inform the research. In addition, monetary compensation can make research engagement more financially feasible for some patient and caregiver partners and can encourage engagement from a broader range of groups. Despite its importance, some participants, like Participant 3, found discussing compensation challenging, creating a barrier to engagement:

I think what helps me the least is the inability to talk about compensation, to talk about expectations… But the amount of work you put in. It should be compensated, and it should be budgeted for, and it should be talked about. We aren’t there yet to talk about it… I’m learning to, but it’s one of the hardest things.

[Participant 3]

Transparent and proactive conversations about compensation allow patient and caregiver partners to make informed decisions about their engagement. Open discussions about compensation also reflect good communication practices, demonstrating respect and value for patient and caregiver partners and affirming that it is reasonable for them to anticipate compensation.

#### Subtheme B: “It Feels Good to Be Involved, but There Has to Be More Than That”—Communicating Impact

Researchers can also demonstrate the value of patient engagement by clearly communicating the impact of patient and caregiver partners’ contributions. This can be done by gathering their feedback, incorporating it into decision-making, and showing how it influenced the project. Participant 4 highlighted the importance of seeing a response to input:

Having an impact means there’s a response to the input that I provide. Maybe it’s a change in how a sentence is worded, maybe it’s adding a couple of questions to a questionnaire, maybe it’s changing some of the layout and the content in an infographic... And if there’s no response, an explanation for why there’s no response.

[Participant 4]

When patient and caregiver partners understand the impact of their contributions, they take more pride in their work and may gain greater confidence in their ability to contribute to future projects, especially those who may have initially doubted their role.

Furthermore, when the outcomes of a research project are personally significant to patient and caregiver partners (eg, related to their own or their loved one’s health), they will likely be invested in sharing the results with the world. Researchers can reinforce the value of patient and caregiver partners by engaging them in knowledge translation activities, co-authoring publications, or explaining how research findings might influence future work. Participant 10 emphasized the importance of tangible impact:

I’ve been doing this for many years… I don’t want to be doing this for nothing… I don’t want to [partner] if I don’t see that there’s really an impact. I mean it makes me feel good, it feels good to be involved, but beyond that, there has to be more than that.

[Participant 10]

#### Subtheme C: “I Don’t See That Impact”—When Patient and Caregiver Partner Value is Not Demonstrated

Barriers to engagement arise when researchers fail to communicate impact, leading patient and caregiver partners to feel undervalued, tokenized, or disconnected. Participant 3 expressed frustration about not knowing how their contributions influenced the project:

I mean, I certainly had an impact on the project because [the researchers] got their funding, [the study] got published. So that was an impact for them, but it was not an impact for me. And I see the need to go so much further in the project, and yet I’ve never been involved since then.

[Participant 3]

Frustration can also arise from mismatched expectations about research timelines and anticipated outcomes. Research-to-practice translation often takes years, which can be discouraging for patient and caregiver partners seeking immediate impact. Researchers must communicate the intended impacts and timelines of their research to patient and caregiver partners. For example, while some studies aim to inform practice or policy, others are designed to build an evidence base and guide future research. By sharing this information at the outset, researchers can ensure patient and caregiver partners’ expectations for research timelines and outcomes are realistic within the project’s scope.

### Theme 3: Psychological Safety

The third theme describes an essential facilitator of patient engagement in research—psychological safety. Psychological safety occurs when members of a team are comfortable and eager to share their ideas, ask questions, and challenge others without fear of being dismissed, ignored, judged, or humiliated [22]. We describe 4 subthemes capturing aspects of the team environment that support psychological safety, including (A) interpersonal skills of team leaders, (B) setting aside time for relationship building, (C) deliberate power-sharing, and (D) physical accessibility. Finally, a fifth subtheme (E) discusses the barriers to a psychologically safe team environment.

#### Subtheme A: “The Principal Investigator Sets the Tone”—Interpersonal Skills of Team Leaders

The interpersonal skills of the principal investigator (PI) are crucial for fostering psychological safety within the team and building reciprocal relationships with patient and caregiver partners. Participants reported positive engagement experiences when their PI demonstrated kindness, warmth, and acceptance. These interpersonal skills set the tone for the entire team and reinforced the value of the patient and caregiver partner role. For example, Participant 10 reflected:

The environment was inclusive from day one and I will credit that to the principal investigator. It was obvious there was a lot of value to what the [patient and caregiver] partners were going to say, and her team just follows that lead…[The PI] sets the tone with all of the people who are working with them.

[Participant 10]

Participants also appreciated when the PI created opportunities for patient and caregiver partners to connect with other researchers and clinicians. This approach helped the patient and caregiver partners to feel more integrated into the broader research and clinical community. While some PIs may naturally possess these relational skills, others may need time and practice to develop them. PIs can consider completing training in leadership and team dynamics or seeking mentorship from other researchers who have successfully facilitated strong teams with patient and caregiver partners. In addition, establishing a team charter or terms of reference can help ensure all members understand the expectations for respectful interactions.

#### Subtheme B: “You Have to Establish a Trusting Relationship Slowly”— Setting Aside Time for Relationship Building

A key component of psychological safety is building trusting relationships. Research teams that dedicated time to informal, friendly discussions were more successful in building trust with patient and caregiver partners. These discussions could take place during brief check-ins at the start of meetings or while sharing meals afterward. Informal settings allowed the research team to connect, learn about each other, and collaborate more effectively. As Participant 5 explained:

I think our collaborations strengthened between the investigator and myself the more we got to know each other, and it wasn’t necessarily in a very formal setting… it’s been those informal chats and her getting to know me and vice versa.

[Participant 5]

Trust is essential for authentic patient engagement, where disagreements are addressed openly and constructively with a sense of curiosity. Respectful discussions foster trust and camaraderie, making it easier to handle challenging conversations. While in-person discussions can enhance trust among team members, they are not always feasible, especially for those in different geographic areas. Alternative methods for building relationships in web-based team settings include organizing one-on-one introductory meetings with the PI or engagement liaison, creating email or messaging groups, dedicating time for check-ins at the beginning of each meeting, or arranging informal coffee calls for team members to get to know each other better.

#### Subtheme C: “Here We Are as People; We’re Going to Work Together”— Deliberate Power-Sharing

Power-sharing practices are another essential element for promoting psychological safety. Effective research teams encouraged all members to leave their degrees and titles at the door and approach meetings with an open mind. This collective approach made patient and caregiver partners feel respected and valued. As Participant 9 explained:

It was the processes that [the researchers] used in regard to bringing the group together and connecting with them that were a big part of the respect. It wasn’t language that was top-down language. It was the ‘we’ language versus the ‘I will’ language. And if they had to make decisions at times they would share them. It was transparent.

[Participant 9]

Power-sharing also involved directly asking patient and caregiver partners for feedback and providing space for quieter voices to contribute. Participant 11 noted, “Every time we meet, [the PI] will say, ‘I’d like to hear from our [patient and caregiver] partners now.” This practice ensured that patient and caregiver partners were regarded as essential contributors, not just names on a paper. It also created a collaborative, respectful environment where new ideas could be shared. Participant 3 emphasized, “When you feel that you’re listened to, you feel that it’s a safer place to speak because it will be accepted.”

Finally, this discussion of power-sharing requires a caveat concerning the word empowerment. For some, this term can be problematic, as it suggests that researchers hold all the power to be shared with patient and caregiver partners. Instead, we believe that researchers should aim to create environments where all team members (both researchers and patient or caregiver partners) can leverage their internal strengths and resources to benefit the research.

#### Subtheme D: “My Patient Experience May Provide Barriers to Participating”—Physical Accessibility

Psychological safety also requires addressing physical accessibility and inclusion. Patient and caregiver partners felt included, respected, and accepted when engagement opportunities were accessible, and accommodations were offered. As Participant 1 shared, “Understanding, from the medical point of view, the restrictions in how and how much people can participate” is crucial. Without accommodations, patient and caregiver partners experienced frustration or feelings of exclusion. Teams that supported patient and caregiver partners’ participation by offering flexible options for meetings, whether in-person, via videoconferencing, or hybrid, made it easier for partners to engage. Providing sufficient time to complete tasks and scheduling work around patient and caregiver partners’ other responsibilities also made their involvement more manageable. In addition, when researchers provided support for technical aspects of the patient or caregiver partner role (eg, completing grant application paperwork) and offered multiple avenues for giving feedback (such as written or verbal options), patient and caregiver partners were better able to complete their tasks and contribute meaningfully.

#### Subtheme E: “There Are Some Unique Challenges to Making That What You Call a Safe Place”—Barriers to Psychological Safety

Despite efforts to promote psychological safety, factors such as low emotional readiness or power imbalances can detract from it. First, participants reflected that if patient and caregiver partners are not emotionally ready to share their lived or living experiences, they may experience negative mental health consequences from their role. Participants emphasized that emotional readiness varies among individuals and can change over time. Our findings suggest that patient and caregiver partners may find it beneficial to consider their emotional readiness when determining if a research project is a good fit for them. In addition, researchers should ensure that mental health resources are available for any team member who may need them.

Power imbalances can also affect psychological safety, especially when a single patient or caregiver partner is involved in a research team. This can lead to isolation and difficulty asking for clarification or sharing one’s perspective. Participant 4 shared, “Many times, I’m the only lay person on the research team… and the language that is used is intimidating.” For patient and caregiver partners, having other non-researcher voices in the room can help balance these dynamics. Finally, patient and caregiver partners may face challenges when working alongside clinicians who are also part of the research team. This concern is especially relevant when a community of clinicians and patients or caregivers is small, and it is more common for partners to be on the same research team as their own health care providers, as Participant 10 noted:

You’re at [the] table now and the doctor who treats your child is there. With that power imbalance are you going to be able to speak up? There’s a lot to consider in those kinds of situations.

[Participant 10]

To address this, researchers should offer opportunities for patient and caregiver partners to contribute anonymously or in separate meetings from clinicians (in addition to full team meetings) to ensure they can provide open and honest feedback about their experiences.

### Theme 4: Community Outreach, Training, and Education

The final theme emphasizes the crucial role of community outreach, training, and education in facilitating patient engagement. Within this theme, three subthemes explore: (A) the importance of community outreach for raising awareness about patient engagement, (B) how training opportunities can support the meaningful engagement of patient and caregiver partners, and (C) how education can help researchers facilitate better engagement.

#### Subtheme A: “We Need to Make People Aware That They Can Be Involved in Research”—The Importance of Community Outreach

Community outreach is vital for ensuring patient engagement is inclusive and reflective of diverse experiences. Many health issues are linked to social determinants, and without broad representation, research risks overlooking valuable perspectives. Participants in our study observed that most patient and caregiver partners came from a narrow demographic, with limited outreach to underrepresented groups. Participant 4 noted:

It’s a high education cohort. It’s a high-income cohort. So, a lot of voices from the general public are probably being filtered out in that environment… Do I think [patient engagement] is inclusive at the moment, I would say no.

[Participant 4]

Typical recruitment methods, such as social media or advertising within established patient and caregiver partner networks, often do not reach individuals outside of the existing patient engagement community. To recruit more diverse patient and caregiver partners or individuals who have not engaged in research before, researchers may need to expand their recruitment toolkit. For example, it is important to use culturally relevant recruitment approaches, collaborate with community leaders, and meet people in familiar environments (eg, libraries and community centers) to build trust and foster strong relationships. When one person from a community engages, they can encourage others to join, thus strengthening community ties to research. As Participant 4 emphasized:

There has to be more of that dedicated outreach that involves both clinicians and existing [patient or caregiver] partners... It has to be planned. It has to be deliberate.

[Participant 4]

#### Subtheme B: “Support Patient and Caregiver Partners in Building Their Research Capacity”—Training Opportunities Contribute to Meaningful Engagement.

Providing training to patient and caregiver partners is a critical strategy for supporting equitable and effective collaboration in research. Although not all partners will require or seek out training, offering such opportunities can help clarify research terminology and expectations, while enhancing partners’ confidence in engaging within research environments. Training may be particularly valuable for first-time patient and caregiver partners or for those entering a new area of research. In the absence of these supports, patient and caregiver partners may encounter barriers that constrain their ability to contribute at their desired capacity or may feel restricted in the scope of their role despite wishing to be more actively involved. As emphasized by Participant 8, adequate preparation is a key foundation for meaningful engagement:

I like being prepared and I like having as much information as I can. So personally, I would have benefited from formal training… maybe some more information about what it looks like to be a [patient or caregiver] partner in terms of communication skills and stuff.

[Participant 8]

Some patient and caregiver partners may also find it helpful to gain familiarity with specific aspects of research, such as ethical review procedures or the publication process. Without this context, changes to the research protocol or delays in progress and publication may become sources of frustration. Training can therefore enhance transparency in decision-making and research workflows by clarifying which aspects of the project are open to patient and caregiver partner influence and which are determined by institutional oversight.

#### Subtheme C: “It Has to Start With Awareness and Education”—Educating Researchers for Better Engagement.

Meaningful engagement with patient and caregiver partners requires specific knowledge and skills, yet many researchers have limited formal training in this area. Without such preparation, engagement efforts may unintentionally become tokenistic. Education offers a way forward by highlighting the value of lived and living experience and by providing practical guidance on how to collaborate respectfully with patient and caregiver partners. While hands-on experience is a valuable learning opportunity, a strong theoretical foundation is also necessary to understand the importance of patient engagement and how it can be effectively implemented. Introducing these concepts early in researchers’ careers, for example, within undergraduate research methods courses and continuing throughout graduate training, can help cultivate a culture where meaningful patient engagement is the norm. As Participant 11 stated:

I think that we need to engage researchers and let them know that getting us involved is going to enhance their research... continuous education of medical researchers, or anybody who’s involved in healthcare research to understand the importance of involving [patient and caregiver] partners right from the beginning.

[Participant 11]

Participants also highlighted the need for more detailed examples of successful patient and caregiver engagement across diverse fields in the published literature. Sharing engagement protocols and reflecting on how engagement shaped research processes and outcomes can contribute to a broader community of practice. Ultimately, education can reinforce that research engagement is grounded in principles of equity and respect for lived and living expertise, rather than being approached as a procedural formality.

### Evaluation of the Engagement Process

The 7 patient-caregiver partners who engaged in the data analysis and co-wrote the results were asked to evaluate the engagement process after the midpoint and end of the study. Evaluations were conducted qualitatively through a 30-minute group discussion and quantitatively using a survey (the Public and Patient Engagement Evaluation Tool [23] at both of these points. All 7 patient-caregiver partners engaged in the group discussions, 6 of the 7 responded to the first survey, and 5 of the 7 responded to the second survey. Overall, 100% of comments suggested the patient-caregiver partners had a positive and meaningful engagement experience. Suggestions for improvement centered around more flexible compensation options (which in this study were constrained by institutional procedures) as well as more relationship-building between the weekday and weekend meeting groups. The qualitative and survey responses from each time point are summarized in Multimedia Appendix 6.

## Discussion

### Principal Findings

This study examined the barriers and facilitators to patient engagement experienced by patient and caregiver partners in the Canadian context. In collaboration with patient-caregiver partners who co-analyzed the data and co-wrote the manuscript, four themes were generated: (1) co-defining roles and expectations; (2) demonstrating the value and impact of engagement; (3) psychological safety; and (4) community outreach, training, and education. These themes align with and provide new insights into existing literature as described below.

### Co-Defining Roles and Expectations

Consistent with our findings, past research highlights patient and caregiver partners’ desire for clarity on team roles, research timeframes, and outcomes [24,25]. However, some researchers may assume patients and caregivers do not wish to be involved in certain tasks or are unsure of how to engage them [26], leading to feelings of exclusion and team tensions [27]. Furthermore, when roles are not clearly defined, patient and caregiver partners have reported feeling overwhelmed by the unpredictable time demands of research [7]. To address these issues, researchers should aim to engage patients and caregivers early in the research process and co-define engagement opportunities throughout the study [7]. As an example of this, Jackson and colleagues [28] appointed a research fellow to oversee all patient engagement activities in their research institute, ensuring patient and caregiver partners were not “overburdened or overlooked” (p.3).

### Demonstrating the Value and Impact of Engagement

Financial remuneration (ie, compensation for time and reimbursement for expenses incurred) is one way to acknowledge patient and caregiver partners’ contributions, symbolizing the value of their lived or living expertise [9] and removing financial barriers to engagement [5]. However, we recognize that remuneration is not always possible due to funding constraints or patient and caregiver partner preferences [6]. In these situations, it is important to work with patient and caregiver partners to identify nonmonetary forms of compensation that are suitable for them (eg, education or skill training, conference attendance, or donations) [29] or to provide remuneration retrospectively after funding is acquired. We also found that compensation alone is insufficient to demonstrate patient and caregiver partners’ value; showing their impact on the research is also essential. Simple strategies, such as using “track changes” in written work, documenting contributions in meetings, and maintaining consistent communication, can demonstrate patient and caregiver partners’ influence on the research.

Our findings and past research both suggest that patient and caregiver partners feel their role is valuable when they understand how research can impact the “real world” (eg, changing future research, policy, or practice) [13]. However, researchers do not always communicate research goals effectively, and some cease communication after a study concludes, leaving patient and caregiver partners uncertain about the project’s impact or success [30]. To address these issues, researchers should engage patient and caregiver partners in knowledge dissemination efforts, such as co-authoring publications [27] and translating findings into accessible formats beyond academic journals [5]. Importantly, co-authorship must be a meaningful, collaborative process, ensuring patient and caregiver partners are fully informed of their role and given opportunities to contribute [31].

### Psychological Safety

Psychologically safe work environments are spaces where research team members feel free to bring their whole selves to work, share ideas, and make mistakes without fear of isolation or exclusion [32]. Effective team relationships, empowerment, and inclusion are important components of psychological safety [32]. Co-writing a team charter or terms of reference at the outset of engagement can help define what a psychologically safe work environment means for the whole team [5,7].

Related research on patient engagement supports our finding that a study’s PI must possess strong relational skills (such as communication, open-mindedness, empathy, and friendliness) to foster meaningful relationships with patient and caregiver partners [10,14]. Dedicating time to relationship-building through “check-in” meetings [7] and group activities [5] can support psychological safety. Monitoring patient and caregiver partners’ experiences through surveys or group discussions can also help address psychological safety concerns as they arise. Power-sharing is another important consideration, as patient and caregiver partners feel encouraged to bring their full selves to a research project when they can contribute their strengths [30] and receive peer support [7]. Researchers must be mindful of group power dynamics; good facilitation practices may involve dedicating specific meetings to gathering feedback from patient and caregiver partners [5] in addition to ensuring that quieter individuals are not overshadowed by more outspoken group members [27]. Finally, when research activities are accessible and accommodating, patient and caregiver partners feel welcomed, valued, and able to engage to their full desired capacity. Researchers should proactively engage in discussions about accessibility, rather than waiting for their research partners to make requests [33]. Patient and caregiver partners’ need for accommodations may also change over time, so accessibility should be an ongoing conversation.

### Community Outreach, Training, and Education

Training and education were key facilitators of patient engagement in this study. Past research shows that patient and caregiver partners want opportunities to be involved in the entire research process and are more willing to take on new roles, such as data analysis, when training is available [24,26]. Researchers can also benefit from training to effectively engage with patient and caregiver partners [14,24,34]. Skills such as communication, integrating patient input [5,26], following engagement guidelines [25], and distinguishing patient engagement from qualitative research [28] can all be honed through training. Multimedia Appendix 7 lists select educational resources identified by the patient-caregiver partners on our authorship team that can support meaningful patient and caregiver engagement in research.

Related literature also notes several barriers to inclusive engagement that require more than education and training to address. These barriers include a lack of community outreach from researchers [24,28], communities’ mistrust of researchers [5], and recruitment strategies that rely only on university or patient and caregiver networks [27]. These barriers may be particularly salient for individuals and communities who have experienced systemic oppression, stigmatization, or historical harms within the health care system and health research. Examples include people with lived experience of substance use or mental health conditions, newcomers to Canada, people experiencing homelessness, and First Nations, Inuit, and Métis Peoples [35]. This underscores the importance of not only supporting researchers as they learn to engage in ways that are respectful, trauma-informed, and culturally safe, but also practicing reflexivity to recognize one’s own social location in relation to the research. Equally important is ensuring that individuals and communities are supported in self-determining their own research directions, priorities, and outcomes [35]. To increase diversity, researchers can provide translation services [7], partner with community leaders or advocates to build trust and guide respectful interactions, intentionally form relationships with underrepresented communities, ensure research priorities are community-driven [36,37], support the development of new patient and caregiver partner groups [6], and create tools to help connect patient and caregiver partners with researchers [25].

### Practical Implications

Throughout the results and discussion, we offer practical recommendations to support the facilitators and address the barriers of patient engagement. To consolidate these recommendations, we have developed an actionable checklist for patient engagement, organized by stages of the research cycle (see Multimedia Appendix 8 ). This checklist serves as a tool for researchers when planning engagement activities and can also help patient and caregiver partners identify supports they can advocate for in their role.

### Strengths and Limitations

This study was conceptualized, co-led, and co-authored by a diverse group of experienced Canadian patient-caregiver partners who were integrated within all aspects of the research process from data collection to qualitative analysis and manuscript writing. As detailed in our engagement evaluation (see Multimedia Appendix 6), several factors contributed to our strong engagement process in this study. These included the co-development of a team charter at the research outset to clarify roles, expectations, and needs; offering flexible meeting times by scheduling both weekday and weekend options; setting aside time for relationship building at the start of each meeting; and providing meeting materials well in advance, along with regular reminders from the patient engagement liaison. These efforts contributed to a respectful and supportive working environment that prioritized relationship-building and accessibility.

Despite these strengths, we encountered several challenges. For example, there was limited flexibility in how patient-caregiver partners could be compensated within our institution. This process created administrative burdens and discomfort for some, particularly around the need to submit Social Insurance Numbers and navigate tax documentation. Another challenge was the unintended division of the patient-caregiver partner team into 2 separate groups based on meeting availability, which limited opportunities for the full group to build collective relationships. Future projects could benefit from scheduling full-group meetings at the beginning, midpoint, and end of the project to foster stronger cross-group cohesion.

### Conclusion

We examined key barriers and facilitators to patient engagement in the Canadian context from the perspectives of experienced patient and caregiver partners. In collaboration with seven patient-caregiver partners, we generated four themes: (1) co-defining roles and expectations, (2) demonstrating the value and impact of engagement, (3) psychological safety, and (4) community outreach, training, and education. When these qualities of engagement were present, meaningful patient engagement was facilitated. When they were absent, barriers to engagement and tokenism arose. To promote the facilitators of engagement and mitigate the barriers, we consolidated our findings into an engagement checklist that is supported by our research findings, the experiences of the patient-caregiver partners on this team, and the patient engagement literature. Researchers, patient partners, and caregiver partners should consider the items in our checklist when planning for their next research partnership endeavor.

## Supplementary material

10.2196/79538 Multimedia Appendix 1 Word cloud depicting the titles patient-caregiver partners in this study identified with.

10.2196/79538 Multimedia Appendix 2 Open sorting instructions for participatory theme elicitation.

10.2196/79538 Multimedia Appendix 3 Data file used in participatory theme elicitation step 4 to conduct the network analysis.

10.2196/79538 Multimedia Appendix 4 Network diagram of participant quotations generated in step 4 of participatory theme elicitation.

10.2196/79538 Multimedia Appendix 5 Image depicting the Miro whiteboard used to conduct data analysis and interpretation in participatory theme elicitation step 5.

10.2196/79538 Multimedia Appendix 6 Patient-caregiver partner evaluations of their engagement in this study.

10.2196/79538 Multimedia Appendix 7 Select patient engagement resources identified by the patient-caregiver partners on our research team.

10.2196/79538 Multimedia Appendix 8 Select considerations when engaging patient-caregiver partners in research studies.

10.2196/79538 Checklist 1 GRIPP2 reporting checklist–short form.

10.2196/79538 Checklist 2 COREQ 32-item checklist.

## Abbreviations

PI: principal investigator
PTE: participatory theme elicitation
