# Supplementary material for: Exploring Patient and Caregiver Perceptions of the Facilitators and Barriers to Patient Engagement in Research: Participatory Qualitative Study
Source: J Particip Med. 2025 Sep 30;17:e79538. doi: 10.2196/79538 (PMC12483476; doi:10.2196/79538)
Supplement: Multimedia Appendix 2 [file jopm-v17-e79538-s002.docx]

| Multimedia Appendix 2: Open sorting instructions for Participatory Theme Elicitation. | |
| --- | --- |
| 1 | Each participant quotation will appear on a separate “sticky note” within your Miro whiteboard. Read through all the sticky notes once while making note of any recurring patterns or ideas that you notice. |
| 2 | Read through the entire dataset again, this time use the “tag” tool in Miro to tag each sticky note with a word/phrase that summarizes the central idea within the participant’s quotation.   - Please use only one tag per quotation. Each quotation must have a tag. - There is no limit to the number of different tags you can create. However, try to re-use tags whenever two or more quotations have similar central ideas. - Each tag should be meaningful and relevant to the data – there should be no “miscellaneous” tag. - Do not worry about using the same tags as other members of the research team. At this stage, it is only important that the tags are meaningful and relevant to you. |
| 3 | After each sticky note has been tagged, drag your cursor to highlight all the sticky notes in your Miro whiteboard. Use the “cluster” tool to group the sticky notes by tag. After completing this step, you will see that all quotations with the same tags are now spatially grouped together. |
| 4 | Review the groups of quotations. If possible, try to consolidate small groups with similar tags into larger groups. Once you feel that each group of quotations speaks to a similar central idea or “theme”, the activity is complete. Create a label for each group of quotations before sharing them with the patient engagement liaison. |
