# Supplementary material for: Exploring Patient and Caregiver Perceptions of the Facilitators and Barriers to Patient Engagement in Research: Participatory Qualitative Study
Source: J Particip Med. 2025 Sep 30;17:e79538. doi: 10.2196/79538 (PMC12483476; doi:10.2196/79538)
Supplement: Multimedia Appendix 3 [file jopm-v17-e79538-s003.docx]

| Topic | Person | Group |
| --- | --- | --- |
| Q35 | 1 | 1 |
| Q43 | 1 | 1 |
| Q4 | 1 | 1 |
| Q13 | 1 | 1 |
| Q5 | 1 | 1 |
| Q82 | 1 | 1 |
| Q36 | 1 | 1 |
| Q68 | 1 | 1 |
| Q1 | 1 | 2 |
| Q48 | 1 | 2 |
| Q54 | 1 | 2 |
| Q63 | 1 | 2 |
| Q58 | 1 | 3 |
| Q71 | 1 | 3 |
| Q76 | 1 | 3 |
| Q84 | 1 | 3 |
| Q23 | 1 | 3 |
| Q15 | 1 | 3 |
| Q25 | 1 | 3 |
| Q79 | 1 | 4 |
| Q87 | 1 | 4 |
| Q57 | 1 | 4 |
| Q38 | 1 | 4 |
| Q50 | 1 | 4 |
| Q27 | 1 | 4 |
| Q20 | 1 | 5 |
| Q47 | 1 | 5 |
| Q32 | 1 | 5 |
| Q80 | 1 | 5 |
| Q19 | 1 | 6 |
| Q21 | 1 | 6 |
| Q51 | 1 | 6 |
| Q74 | 1 | 6 |
| Q75 | 1 | 6 |
| Q77 | 1 | 6 |
| Q16 | 1 | 7 |
| Q34 | 1 | 7 |
| Q29 | 1 | 7 |
| Q65 | 1 | 7 |
| Q30 | 1 | 7 |
| Q78 | 1 | 7 |
| Q39 | 1 | 7 |
| Q11 | 1 | 8 |
| Q24 | 1 | 8 |
| Q37 | 1 | 8 |
| Q46 | 1 | 8 |
| Q86 | 1 | 8 |
| Q12 | 1 | 8 |
| Q90 | 1 | 8 |
| Q18 | 1 | 8 |
| Q17 | 1 | 8 |
| Q22 | 1 | 8 |
| Q40 | 1 | 9 |
| Q55 | 1 | 9 |
| Q62 | 1 | 9 |
| Q72 | 1 | 9 |
| Q73 | 1 | 9 |
| Q85 | 1 | 9 |
| Q8 | 1 | 9 |
| Q33 | 1 | 10 |
| Q44 | 1 | 10 |
| Q67 | 1 | 10 |
| Q83 | 1 | 10 |
| Q89 | 1 | 10 |
| Q52 | 1 | 11 |
| Q56 | 1 | 11 |
| Q61 | 1 | 11 |
| Q64 | 1 | 11 |
| Q66 | 1 | 11 |
| Q81 | 1 | 11 |
| Q9 | 1 | 11 |
| Q49 | 1 | 11 |
| Q2 | 1 | 11 |
| Q7 | 1 | 11 |
| Q6 | 1 | 11 |
| Q88 | 1 | 12 |
| Q42 | 1 | 12 |
| Q59 | 1 | 12 |
| Q3 | 1 | 12 |
| Q41 | 1 | 12 |
| Q14 | 1 | 12 |
| Q69 | 1 | 12 |
| Q70 | 1 | 12 |
| Q10 | 1 | 13 |
| Q31 | 1 | 13 |
| Q53 | 1 | 13 |
| Q60 | 1 | 13 |
| Q45 | 1 | 13 |
| Q26 | 1 | 13 |
| Q28 | 1 | 13 |
| Q40 | 2 | 1 |
| Q13 | 2 | 1 |
| Q50 | 2 | 1 |
| Q14 | 2 | 1 |
| Q88 | 2 | 1 |
| Q4 | 2 | 1 |
| Q19 | 2 | 1 |
| Q47 | 2 | 1 |
| Q11 | 2 | 1 |
| Q12 | 2 | 1 |
| Q35 | 2 | 1 |
| Q15 | 2 | 1 |
| Q7 | 2 | 1 |
| Q31 | 2 | 1 |
| Q59 | 2 | 1 |
| Q86 | 2 | 1 |
| Q5 | 2 | 1 |
| Q24 | 2 | 1 |
| Q89 | 2 | 1 |
| Q23 | 2 | 1 |
| Q3 | 2 | 1 |
| Q32 | 2 | 1 |
| Q21 | 2 | 1 |
| Q22 | 2 | 1 |
| Q9 | 2 | 1 |
| Q18 | 2 | 1 |
| Q37 | 2 | 1 |
| Q80 | 2 | 1 |
| Q79 | 2 | 1 |
| Q62 | 2 | 1 |
| Q35 | 2 | 1 |
| Q87 | 2 | 1 |
| Q51 | 2 | 1 |
| Q27 | 2 | 1 |
| Q25 | 2 | 1 |
| Q34 | 2 | 1 |
| Q17 | 2 | 1 |
| Q66 | 2 | 1 |
| Q71 | 2 | 1 |
| Q57 | 2 | 1 |
| Q52 | 2 | 1 |
| Q55 | 2 | 1 |
| Q58 | 2 | 1 |
| Q46 | 2 | 1 |
| Q49 | 2 | 1 |
| Q53 | 2 | 1 |
| Q75 | 2 | 1 |
| Q76 | 2 | 1 |
| Q70 | 2 | 1 |
| Q77 | 2 | 1 |
| Q90 | 2 | 1 |
| Q72 | 2 | 1 |
| Q74 | 2 | 1 |
| Q1 | 2 | 2 |
| Q33 | 2 | 2 |
| Q63 | 2 | 2 |
| Q54 | 2 | 2 |
| Q6 | 2 | 2 |
| Q73 | 2 | 2 |
| Q48 | 2 | 2 |
| Q83 | 2 | 2 |
| Q64 | 2 | 2 |
| Q82 | 2 | 2 |
| Q36 | 2 | 2 |
| Q42 | 2 | 2 |
| Q65 | 2 | 2 |
| Q68 | 2 | 2 |
| Q20 | 2 | 3 |
| Q78 | 2 | 3 |
| Q56 | 2 | 3 |
| Q61 | 2 | 3 |
| Q81 | 2 | 3 |
| Q60 | 2 | 3 |
| Q2 | 2 | 3 |
| Q28 | 2 | 3 |
| Q43 | 2 | 3 |
| Q26 | 2 | 3 |
| Q45 | 2 | 3 |
| Q67 | 2 | 3 |
| Q44 | 2 | 3 |
| Q30 | 2 | 4 |
| Q38 | 2 | 4 |
| Q69 | 2 | 4 |
| Q85 | 2 | 4 |
| Q10 | 2 | 4 |
| Q39 | 2 | 4 |
| Q29 | 2 | 4 |
| Q8 | 2 | 4 |
| Q16 | 2 | 4 |
| Q84 | 2 | 4 |
| Q60 | 3 | 1 |
| Q18 | 3 | 1 |
| Q23 | 3 | 1 |
| Q6 | 3 | 1 |
| Q48 | 3 | 1 |
| Q5 | 3 | 1 |
| Q3 | 3 | 1 |
| Q59 | 3 | 1 |
| Q88 | 3 | 1 |
| Q27 | 3 | 1 |
| Q45 | 3 | 1 |
| Q41 | 3 | 1 |
| Q14 | 3 | 1 |
| Q42 | 3 | 1 |
| Q43 | 3 | 1 |
| Q19 | 3 | 1 |
| Q90 | 3 | 1 |
| Q35 | 3 | 1 |
| Q50 | 3 | 2 |
| Q38 | 3 | 2 |
| Q79 | 3 | 2 |
| Q57 | 3 | 2 |
| Q51 | 3 | 3 |
| Q11 | 3 | 3 |
| Q80 | 3 | 3 |
| Q71 | 3 | 3 |
| Q4 | 3 | 3 |
| Q21 | 3 | 3 |
| Q13 | 3 | 3 |
| Q72 | 3 | 3 |
| Q52 | 3 | 3 |
| Q15 | 3 | 3 |
| Q74 | 3 | 3 |
| Q25 | 3 | 3 |
| Q10 | 3 | 3 |
| Q87 | 3 | 3 |
| Q17 | 3 | 3 |
| Q75 | 3 | 3 |
| Q34 | 3 | 3 |
| Q24 | 3 | 3 |
| Q12 | 3 | 3 |
| Q77 | 3 | 3 |
| Q7 | 3 | 3 |
| Q66 | 3 | 3 |
| Q16 | 3 | 3 |
| Q36 | 3 | 3 |
| Q40 | 3 | 3 |
| Q9 | 3 | 3 |
| Q58 | 3 | 3 |
| Q8 | 3 | 3 |
| Q39 | 3 | 3 |
| Q20 | 3 | 4 |
| Q78 | 3 | 4 |
| Q56 | 3 | 4 |
| Q64 | 3 | 4 |
| Q73 | 3 | 4 |
| Q2 | 3 | 4 |
| Q81 | 3 | 4 |
| Q61 | 3 | 4 |
| Q44 | 3 | 5 |
| Q70 | 3 | 5 |
| Q29 | 3 | 5 |
| Q67 | 3 | 5 |
| Q83 | 3 | 5 |
| Q47 | 3 | 5 |
| Q69 | 3 | 5 |
| Q86 | 3 | 5 |
| Q76 | 3 | 5 |
| Q53 | 3 | 5 |
| Q46 | 3 | 5 |
| Q49 | 3 | 5 |
| Q85 | 3 | 5 |
| Q63 | 3 | 5 |
| Q62 | 3 | 5 |
| Q89 | 3 | 5 |
| Q55 | 3 | 5 |
| Q37 | 3 | 6 |
| Q1 | 3 | 6 |
| Q32 | 3 | 6 |
| Q82 | 3 | 6 |
| Q28 | 3 | 6 |
| Q33 | 3 | 6 |
| Q54 | 3 | 6 |
| Q68 | 3 | 6 |
| Q65 | 3 | 6 |
| Q31 | 3 | 6 |
| Q30 | 3 | 6 |
| Q26 | 3 | 6 |
| Q22 | 3 | 6 |
| Q84 | 3 | 6 |
| Q1 | 4 | 1 |
| Q63 | 4 | 1 |
| Q16 | 4 | 1 |
| Q49 | 4 | 1 |
| Q66 | 4 | 1 |
| Q13 | 4 | 1 |
| Q6 | 4 | 1 |
| Q32 | 4 | 2 |
| Q40 | 4 | 2 |
| Q25 | 4 | 2 |
| Q72 | 4 | 2 |
| Q80 | 4 | 3 |
| Q47 | 4 | 3 |
| Q20 | 4 | 3 |
| Q41 | 4 | 4 |
| Q39 | 4 | 4 |
| Q18 | 4 | 5 |
| Q90 | 4 | 5 |
| Q64 | 4 | 6 |
| Q46 | 4 | 6 |
| Q68 | 4 | 7 |
| Q36 | 4 | 7 |
| Q7 | 4 | 8 |
| Q62 | 4 | 8 |
| Q12 | 4 | 8 |
| Q54 | 4 | 8 |
| Q48 | 4 | 8 |
| Q4 | 4 | 8 |
| Q52 | 4 | 9 |
| Q9 | 4 | 9 |
| Q2 | 4 | 9 |
| Q69 | 4 | 10 |
| Q70 | 4 | 10 |
| Q5 | 4 | 10 |
| Q88 | 4 | 11 |
| Q59 | 4 | 11 |
| Q42 | 4 | 11 |
| Q14 | 4 | 11 |
| Q3 | 4 | 11 |
| Q37 | 4 | 12 |
| Q85 | 4 | 12 |
| Q8 | 4 | 12 |
| Q26 | 4 | 13 |
| Q35 | 4 | 13 |
| Q10 | 4 | 13 |
| Q19 | 4 | 13 |
| Q79 | 4 | 14 |
| Q57 | 4 | 14 |
| Q50 | 4 | 14 |
| Q38 | 4 | 14 |
| Q65 | 4 | 15 |
| Q78 | 4 | 15 |
| Q30 | 4 | 15 |
| Q17 | 4 | 15 |
| Q82 | 4 | 16 |
| Q81 | 4 | 16 |
| Q73 | 4 | 16 |
| Q61 | 4 | 16 |
| Q56 | 4 | 16 |
| Q31 | 4 | 16 |
| Q29 | 4 | 16 |
| Q28 | 4 | 16 |
| Q77 | 4 | 17 |
| Q51 | 4 | 17 |
| Q34 | 4 | 17 |
| Q21 | 4 | 17 |
| Q22 | 4 | 18 |
| Q86 | 4 | 18 |
| Q11 | 4 | 18 |
| Q87 | 4 | 19 |
| Q74 | 4 | 19 |
| Q75 | 4 | 19 |
| Q55 | 4 | 19 |
| Q58 | 4 | 19 |
| Q27 | 4 | 19 |
| Q24 | 4 | 19 |
| Q23 | 4 | 19 |
| Q15 | 4 | 19 |
| Q89 | 4 | 20 |
| Q83 | 4 | 20 |
| Q67 | 4 | 20 |
| Q53 | 4 | 20 |
| Q60 | 4 | 20 |
| Q45 | 4 | 20 |
| Q44 | 4 | 20 |
| Q43 | 4 | 20 |
| Q33 | 4 | 20 |
| Q71 | 4 | 21 |
| Q84 | 4 | 21 |
| Q76 | 4 | 21 |
| Q42 | 5 | 1 |
| Q41 | 5 | 1 |
| Q39 | 5 | 2 |
| Q37 | 5 | 2 |
| Q19 | 5 | 3 |
| Q28 | 5 | 3 |
| Q43 | 5 | 3 |
| Q45 | 5 | 3 |
| Q44 | 5 | 3 |
| Q33 | 5 | 3 |
| Q83 | 5 | 3 |
| Q89 | 5 | 3 |
| Q60 | 5 | 3 |
| Q8 | 5 | 4 |
| Q55 | 5 | 4 |
| Q58 | 5 | 4 |
| Q87 | 5 | 4 |
| Q40 | 5 | 4 |
| Q73 | 5 | 4 |
| Q24 | 5 | 4 |
| Q85 | 5 | 4 |
| Q86 | 5 | 4 |
| Q72 | 5 | 4 |
| Q51 | 5 | 4 |
| Q62 | 5 | 4 |
| Q38 | 5 | 4 |
| Q9 | 5 | 5 |
| Q75 | 5 | 5 |
| Q52 | 5 | 5 |
| Q90 | 5 | 5 |
| Q15 | 5 | 5 |
| Q12 | 5 | 5 |
| Q13 | 5 | 5 |
| Q70 | 5 | 5 |
| Q71 | 5 | 5 |
| Q61 | 5 | 5 |
| Q23 | 5 | 5 |
| Q27 | 5 | 5 |
| Q49 | 5 | 5 |
| Q25 | 5 | 5 |
| Q22 | 5 | 5 |
| Q74 | 5 | 5 |
| Q16 | 5 | 5 |
| Q20 | 5 | 5 |
| Q59 | 5 | 5 |
| Q11 | 5 | 5 |
| Q6 | 5 | 5 |
| Q81 | 5 | 6 |
| Q56 | 5 | 6 |
| Q2 | 5 | 6 |
| Q64 | 5 | 6 |
| Q50 | 5 | 7 |
| Q57 | 5 | 7 |
| Q79 | 5 | 7 |
| Q48 | 5 | 8 |
| Q66 | 5 | 8 |
| Q54 | 5 | 8 |
| Q63 | 5 | 8 |
| Q1 | 5 | 8 |
| Q53 | 5 | 9 |
| Q80 | 5 | 9 |
| Q47 | 5 | 9 |
| Q21 | 5 | 9 |
| Q32 | 5 | 9 |
| Q18 | 5 | 9 |
| Q34 | 5 | 10 |
| Q82 | 5 | 10 |
| Q77 | 5 | 10 |
| Q78 | 5 | 10 |
| Q5 | 5 | 10 |
| Q17 | 5 | 10 |
| Q46 | 5 | 10 |
| Q4 | 5 | 10 |
| Q29 | 5 | 10 |
| Q30 | 5 | 10 |
| Q65 | 5 | 10 |
| Q36 | 5 | 11 |
| Q26 | 5 | 11 |
| Q10 | 5 | 11 |
| Q31 | 5 | 11 |
| Q68 | 5 | 11 |
| Q84 | 5 | 12 |
| Q67 | 5 | 12 |
| Q7 | 5 | 12 |
| Q76 | 5 | 12 |
| Q35 | 5 | 12 |
| Q3 | 5 | 13 |
| Q88 | 5 | 13 |
| Q69 | 5 | 13 |
| Q14 | 5 | 13 |
| Q55 | 6 | 1 |
| Q62 | 6 | 1 |
| Q25 | 6 | 1 |
| Q8 | 6 | 1 |
| Q76 | 6 | 1 |
| Q72 | 6 | 1 |
| Q71 | 6 | 1 |
| Q77 | 6 | 1 |
| Q85 | 6 | 1 |
| Q40 | 6 | 1 |
| Q20 | 6 | 1 |
| Q73 | 6 | 1 |
| Q58 | 6 | 1 |
| Q74 | 6 | 1 |
| Q84 | 6 | 1 |
| Q2 | 6 | 2 |
| Q83 | 6 | 2 |
| Q63 | 6 | 2 |
| Q64 | 6 | 2 |
| Q66 | 6 | 2 |
| Q52 | 6 | 2 |
| Q6 | 6 | 2 |
| Q16 | 6 | 2 |
| Q1 | 6 | 2 |
| Q7 | 6 | 2 |
| Q48 | 6 | 2 |
| Q65 | 6 | 3 |
| Q10 | 6 | 3 |
| Q17 | 6 | 3 |
| Q78 | 6 | 3 |
| Q18 | 6 | 3 |
| Q36 | 6 | 3 |
| Q54 | 6 | 3 |
| Q35 | 6 | 3 |
| Q46 | 6 | 3 |
| Q31 | 6 | 3 |
| Q30 | 6 | 3 |
| Q22 | 6 | 3 |
| Q68 | 6 | 3 |
| Q24 | 6 | 3 |
| Q13 | 6 | 3 |
| Q4 | 6 | 3 |
| Q41 | 6 | 4 |
| Q27 | 6 | 4 |
| Q3 | 6 | 4 |
| Q87 | 6 | 4 |
| Q19 | 6 | 4 |
| Q34 | 6 | 5 |
| Q21 | 6 | 5 |
| Q15 | 6 | 5 |
| Q39 | 6 | 5 |
| Q53 | 6 | 5 |
| Q37 | 6 | 5 |
| Q23 | 6 | 5 |
| Q75 | 6 | 5 |
| Q33 | 6 | 6 |
| Q44 | 6 | 6 |
| Q67 | 6 | 6 |
| Q90 | 6 | 6 |
| Q89 | 6 | 6 |
| Q14 | 6 | 7 |
| Q26 | 6 | 7 |
| Q28 | 6 | 7 |
| Q12 | 6 | 7 |
| Q59 | 6 | 7 |
| Q45 | 6 | 7 |
| Q42 | 6 | 7 |
| Q43 | 6 | 7 |
| Q88 | 6 | 7 |
| Q60 | 6 | 7 |
| Q56 | 6 | 8 |
| Q86 | 6 | 8 |
| Q81 | 6 | 8 |
| Q61 | 6 | 8 |
| Q49 | 6 | 9 |
| Q9 | 6 | 9 |
| Q38 | 6 | 9 |
| Q29 | 6 | 9 |
| Q82 | 6 | 9 |
| Q5 | 6 | 9 |
| Q69 | 6 | 9 |
| Q70 | 6 | 9 |
| Q11 | 6 | 9 |
| Q32 | 6 | 10 |
| Q51 | 6 | 10 |
| Q47 | 6 | 10 |
| Q80 | 6 | 10 |
| Q57 | 6 | 11 |
| Q50 | 6 | 11 |
| Q79 | 6 | 11 |
| Q85 | 7 | 1 |
| Q1 | 7 | 1 |
| Q84 | 7 | 1 |
| Q4 | 7 | 2 |
| Q5 | 7 | 2 |
| Q43 | 7 | 2 |
| Q16 | 7 | 3 |
| Q44 | 7 | 3 |
| Q30 | 7 | 3 |
| Q69 | 7 | 3 |
| Q67 | 7 | 3 |
| Q10 | 7 | 4 |
| Q36 | 7 | 4 |
| Q68 | 7 | 4 |
| Q39 | 7 | 5 |
| Q8 | 7 | 5 |
| Q48 | 7 | 6 |
| Q54 | 7 | 6 |
| Q26 | 7 | 7 |
| Q45 | 7 | 7 |
| Q28 | 7 | 7 |
| Q31 | 7 | 7 |
| Q61 | 7 | 7 |
| Q20 | 7 | 7 |
| Q56 | 7 | 7 |
| Q81 | 7 | 7 |
| Q89 | 7 | 7 |
| Q73 | 7 | 7 |
| Q13 | 7 | 8 |
| Q24 | 7 | 8 |
| Q41 | 7 | 8 |
| Q17 | 7 | 8 |
| Q18 | 7 | 8 |
| Q29 | 7 | 8 |
| Q70 | 7 | 8 |
| Q42 | 7 | 8 |
| Q46 | 7 | 8 |
| Q65 | 7 | 8 |
| Q90 | 7 | 8 |
| Q78 | 7 | 8 |
| Q64 | 7 | 8 |
| Q83 | 7 | 8 |
| Q82 | 7 | 8 |
| Q38 | 7 | 9 |
| Q35 | 7 | 9 |
| Q27 | 7 | 9 |
| Q57 | 7 | 9 |
| Q59 | 7 | 9 |
| Q21 | 7 | 9 |
| Q14 | 7 | 9 |
| Q19 | 7 | 9 |
| Q34 | 7 | 9 |
| Q87 | 7 | 9 |
| Q79 | 7 | 9 |
| Q60 | 7 | 9 |
| Q50 | 7 | 9 |
| Q9 | 7 | 10 |
| Q32 | 7 | 10 |
| Q23 | 7 | 10 |
| Q15 | 7 | 10 |
| Q3 | 7 | 10 |
| Q55 | 7 | 10 |
| Q58 | 7 | 10 |
| Q47 | 7 | 10 |
| Q49 | 7 | 10 |
| Q66 | 7 | 10 |
| Q40 | 7 | 10 |
| Q51 | 7 | 10 |
| Q52 | 7 | 10 |
| Q63 | 7 | 10 |
| Q76 | 7 | 10 |
| Q77 | 7 | 10 |
| Q80 | 7 | 10 |
| Q62 | 7 | 10 |
| Q72 | 7 | 10 |
| Q71 | 7 | 10 |
| Q2 | 7 | 11 |
| Q25 | 7 | 11 |
| Q88 | 7 | 11 |
| Q33 | 7 | 11 |
| Q22 | 7 | 12 |
| Q11 | 7 | 12 |
| Q12 | 7 | 12 |
| Q53 | 7 | 12 |
| Q37 | 7 | 12 |
| Q75 | 7 | 12 |
| Q74 | 7 | 12 |
| Q86 | 7 | 12 |
| Q7 | 7 | 13 |
| Q6 | 7 | 13 |
| Q4 | 8 | 1 |
| Q13 | 8 | 1 |
| Q22 | 8 | 1 |
| Q54 | 8 | 1 |
| Q58 | 8 | 1 |
| Q62 | 8 | 1 |
| Q71 | 8 | 1 |
| Q3 | 8 | 2 |
| Q5 | 8 | 2 |
| Q14 | 8 | 2 |
| Q12 | 8 | 2 |
| Q27 | 8 | 2 |
| Q40 | 8 | 2 |
| Q50 | 8 | 2 |
| Q53 | 8 | 2 |
| Q59 | 8 | 2 |
| Q57 | 8 | 2 |
| Q52 | 8 | 2 |
| Q75 | 8 | 2 |
| Q72 | 8 | 2 |
| Q77 | 8 | 2 |
| Q87 | 8 | 2 |
| Q10 | 8 | 3 |
| Q9 | 8 | 3 |
| Q8 | 8 | 3 |
| Q33 | 8 | 3 |
| Q19 | 8 | 4 |
| Q7 | 8 | 5 |
| Q48 | 8 | 5 |
| Q70 | 8 | 5 |
| Q82 | 8 | 5 |
| Q7 | 8 | 6 |
| Q17 | 8 | 6 |
| Q29 | 8 | 6 |
| Q56 | 8 | 6 |
| Q24 | 8 | 7 |
| Q23 | 8 | 7 |
| Q28 | 8 | 7 |
| Q39 | 8 | 8 |
| Q36 | 8 | 8 |
| Q65 | 8 | 8 |
| Q64 | 8 | 8 |
| Q84 | 8 | 8 |
| Q44 | 8 | 9 |
| Q83 | 8 | 9 |
| Q89 | 8 | 9 |
| Q43 | 8 | 10 |
| Q69 | 8 | 11 |
| Q1 | 8 | 12 |
| Q16 | 8 | 12 |
| Q42 | 8 | 12 |
| Q6 | 8 | 13 |
| Q2 | 8 | 13 |
| Q15 | 8 | 13 |
| Q80 | 8 | 13 |
| Q68 | 8 | 14 |
| Q20 | 8 | 14 |
| Q18 | 8 | 14 |
| Q78 | 8 | 14 |
| Q81 | 8 | 14 |
| Q11 | 8 | 15 |
| Q30 | 8 | 15 |
| Q25 | 8 | 16 |
| Q35 | 8 | 16 |
| Q49 | 8 | 16 |
| Q47 | 8 | 16 |
| Q55 | 8 | 16 |
| Q60 | 8 | 16 |
| Q76 | 8 | 16 |
| Q74 | 8 | 16 |
| Q79 | 8 | 16 |
| Q88 | 8 | 16 |
| Q26 | 8 | 17 |
| Q21 | 8 | 17 |
| Q34 | 8 | 17 |
| Q37 | 8 | 17 |
| Q32 | 8 | 17 |
| Q46 | 8 | 17 |
| Q45 | 8 | 17 |
| Q41 | 8 | 17 |
| Q61 | 8 | 17 |
| Q90 | 8 | 17 |
| Q38 | 8 | 18 |
| Q31 | 8 | 18 |
| Q51 | 8 | 18 |
| Q86 | 8 | 18 |
| Q85 | 8 | 18 |
| Q66 | 8 | 19 |
| Q63 | 8 | 19 |
| Q73 | 8 | 19 |
| Q67 | 8 | 20 |
