# Supplementary material for: Exploring Patient and Caregiver Perceptions of the Facilitators and Barriers to Patient Engagement in Research: Participatory Qualitative Study
Source: J Particip Med. 2025 Sep 30;17:e79538. doi: 10.2196/79538 (PMC12483476; doi:10.2196/79538)

Multimedia Appendix 4: Network diagram of participant quotations generated in step 4 of Participatory Theme Elicitation


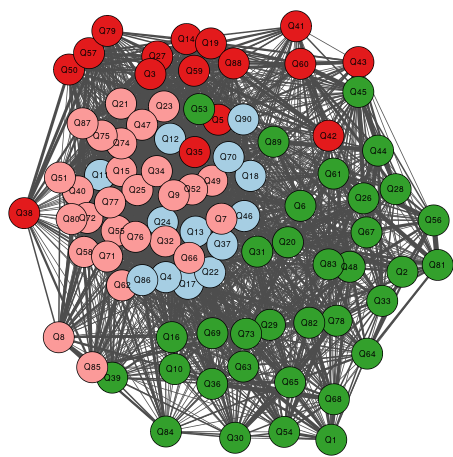

Supplement: Multimedia Appendix 4 [file jopm-v17-e79538-s004.docx]
