# Supplementary material for: Exploring Patient and Caregiver Perceptions of the Facilitators and Barriers to Patient Engagement in Research: Participatory Qualitative Study
Source: J Particip Med. 2025 Sep 30;17:e79538. doi: 10.2196/79538 (PMC12483476; doi:10.2196/79538)
Supplement: Multimedia Appendix 5 [file jopm-v17-e79538-s005.docx]

Multimedia Appendix 5: Image depicting the Miro whiteboard used to conduct data analysis and interpretation in Participatory Theme Elicitation Step 5

**
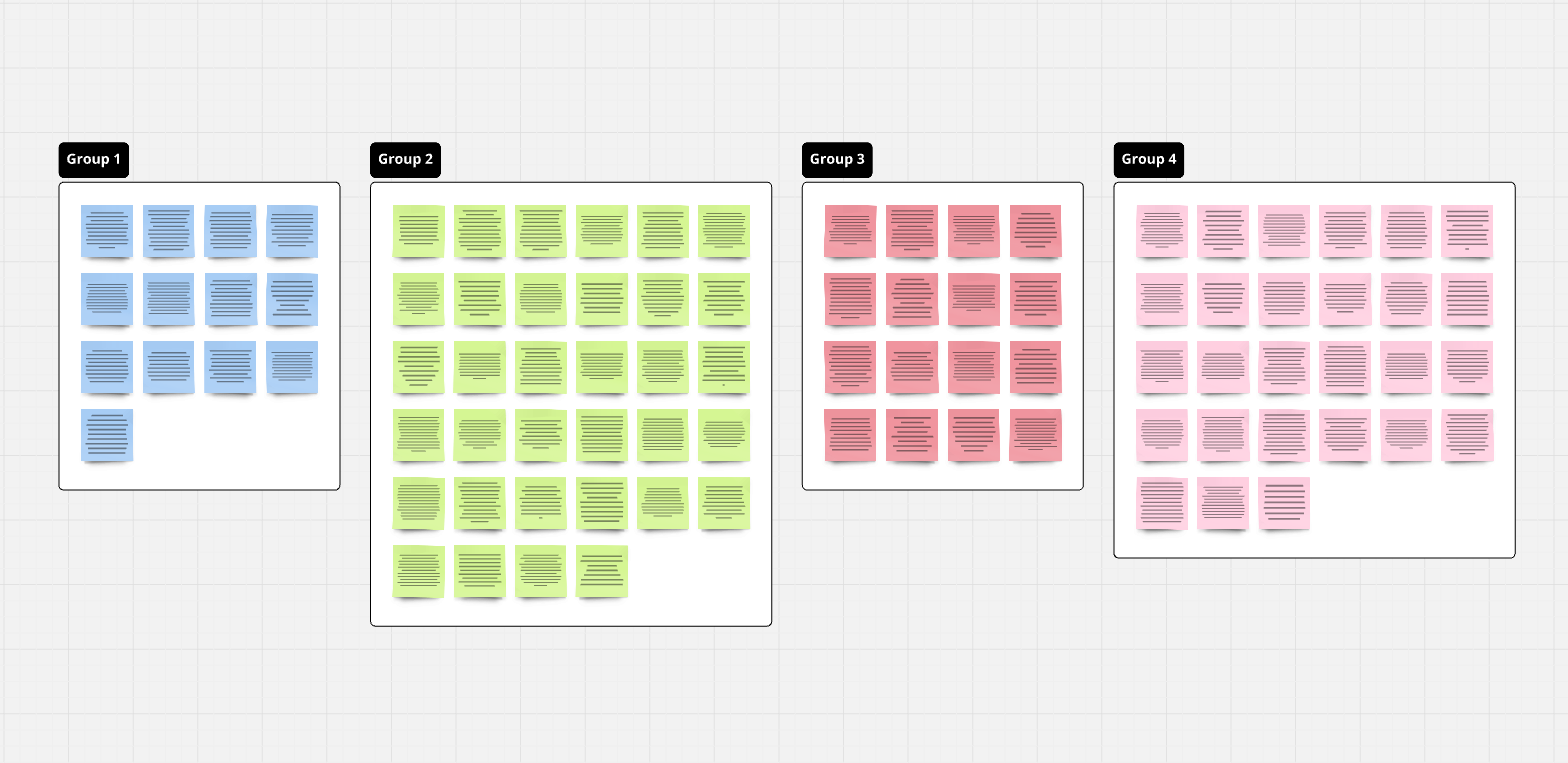
**
