# Supplementary material for: Exploring Patient and Caregiver Perceptions of the Facilitators and Barriers to Patient Engagement in Research: Participatory Qualitative Study
Source: J Particip Med. 2025 Sep 30;17:e79538. doi: 10.2196/79538 (PMC12483476; doi:10.2196/79538)
Supplement: Multimedia Appendix 6 [file jopm-v17-e79538-s006.docx]

| Multimedia Appendix 6: Patient-caregiver partner evaluations of their engagement in the present study  *Summary of Responses to the Public and Patient Engagement Evaluation Tool (PPEET)* | | |
| --- | --- | --- |
| PPEET Item | Evaluation 1 (midpoint)  N = 6 responses | Evaluation 2 (end-of-study)  N = 5 responses |
| 1. I had a clear understanding of the purpose of the study | 100% Agree/Strongly Agree | 100% Agree/Strongly Agree |
| 2. The supports I needed to participate were available | 100% Agree/Strongly Agree | 100% Agree/Strongly Agree |
| 3. I had enough information to contribute to the topics being discussed | 100% Agree/Strongly Agree | 100% Agree/Strongly Agree |
| 4. I was able to express my views freely | 100% Agree/Strongly Agree | 100% Agree/Strongly Agree |
| 5. I felt that my views were heard | 100% Agree/Strongly Agree | 100% Agree/Strongly Agree |
| 6. A wide range of views on the topics we discussed were shared | 100% Agree/Strongly Agree | 100% Agree/Strongly Agree |
| 7. The other patient partners involved in the study represented a broad range of perspectives | 100% Agree/Strongly Agree | 100% Agree/Strongly Agree |
| 8. I think that our study achieved its objectives | 100% Agree/Strongly Agree | 100% Agree/Strongly Agree |
| 9. I am confident that the input I provided will be accurately and truly reflected in the final write-up of the research project | 100% Agree/Strongly Agree | 100% Agree/Strongly Agree |
| 10. I think the input I provided made a difference to the research project | 100% Agree/Strongly Agree | 100% Agree/Strongly Agree |
| 11. As a result of my work on this study, I am better informed about the barriers and facilitators to patient engagement | 100% Agree/Strongly Agree | 100% Agree/Strongly Agree |
| 12. Overall I was satisfied with this engagement initiative | 100% Agree/Strongly Agree | 100% Agree/Strongly Agree |
| 13. Engaging in this project was a good use of my time | 100% Agree/Strongly Agree | 100% Agree/Strongly Agree |

| **Online Resource 4.**  *Qualitative Evaluation Responses* | | |
| --- | --- | --- |
| Question | Responses (midpoint) | Responses (end-of-study) |
| *Is there anything else you would like to share about how your engagement in the project was or was not supported?* | *[Facilitator] was very approachable...made things great for the PP*  *[Facilitator] was wonderful. She's incredibly organized and articulate in all her emails/asks. I think she was critical in making sure that our engagement was productive and meaningful. I think making us employees of [the University] also helped me feel like I was contributing.*  *Fully engaged and pre engagement interview very good. Make sure all team members offer their thoughts, as in don't let the extroverts take over. Flexibility with time for meetings, agenda for meetings excellent. Always positive.*  *Request for direct payment as Independent Contractor rather than onboarded as staff requiring the sharing of my SIN. It was too cumbersome for this type of work. A T4 for ~$500 seems inefficient.*  *I felt completely supported. You had a challenge keeping everyone on task during some meetings and you handled it exceptionally well.* | *Great support, we had two weekly options to attend meetings and I believe both groups worked very well together. I preferred to attend the Sunday group as it worked better with my schedule.*  *I felt highly supported and engaged and really appreciated knowing how to contribute. What was expected of me was outlined clearly so that I felt like I added value to the project.*  *Partnering in this truly was an exceptional experience in terms of support, transparency, and enthusiasm from [the facilitator]. It felt more like she was one of us, i.e. more than an equal partnership.* |
| *Is there anything else you would like us to know about your ability to share your views during the study?* | *Great team...lots of perspectives*  *I was in a group of people I would likely have never chosen to be in which I actually loved. I really enjoy being exposed to views that I don't normally come across in my daily life and being challenged to think outside of my comfort zone.*  *I overshare, sometimes the quieter ones don't speak up. need encouragement. I have heard some patient partners say they don't like being called upon and if have something to say they will. Could be checked where people are at with that, are they ok being asked what they think, or will they speak up if have something to add.*  *Everyone was encouraged to share and interact* | *As a group we idea tossed and fed of off each other’s views and opinions. We had very good discussions and different perspectives that worked well together. We had both rural and urban representation and a pan Canadian representation. We represented both patients, families and caregivers.*  *I love being stretched in terms of engaging with people who aren't usually in my circle and loved that no one was defensive or easily offended. I felt really comfortable even though we all came from such different perspectives.* |
| *Is there anything else we should know about your ability to impact the study or the impact you hope the study will have?* | *I hope it will inspire other researchers to include more patient partners. I don't know how [facilitator] felt - if it was way more work to include so many people who aren't researchers - but if not, she should really write up her experience to share with other researchers on best practices to engage patient partners.*  *Hoping it will change practices that negatively impact patient partner engagement, encourage conversations among principal investigators regarding best practices, increase awareness of patient-oriented-research and how to become a patient partner and what equal partnership looks like.*  *I think it will have a positive impact on patient/researcher relationships and the engagement process overall* | *It was a great experience.* |
| *In your opinion, what went well about our work together?* | *The diversity in the patient partners and the support from [Facilitator]*  *The facilitator!!!! And the team she assembled*  *[The facilitator}! The carefully thought-out way you included patient partners. Our time wasn't wasted. The whole process was efficient - the timeline was quick and it was followed and it wasn't dragged on indefinitely. I knew when meetings were, what they were going to be about and when I could expect compensation. I knew who to contact, that I could miss a meeting and it would be OK, and I didn't feel like I had imposter syndrome because I wasn't the single patient partner in the group - others were experienced but they were also humble about expressing their lack of knowledge so it made me feel comfortable to say when I didn't understand something too.*  *The process was excellent. Although the quotes didn't represent any aha moments, I feel like the organization and categorizing of themes etc is unique in many ways.*  *[The person] leading it! What a facilitator!*  *Your willingness to learn alongside us and your facilitation skills.* | *It was a safe space and Sasha did a great job facilitating the discussions.*  *Everything went well*  *I think everything went really well.*  *[The facilitator] was an incredible facilitator and project manager. She kept the project moving, and was organized about when we would meet and what we would do. She had an obvious plan from the start and knew exactly how to engage us "non-research" people in a way where we could effectively contribute to research.*  *There was such a good atmosphere of trust among the group. I believe everyone felt comfortable expressing their thoughts and ideas. A good example of relational engagement.* |
| *In your opinion, what could have been improved about our work together?* | *maybe a practice round of classifying the different quote before doing the real work*  *The weekly/bi-weekly timesheets are a bit of a pain but it really wasn't that bad. Maybe having some of us as a part of the original interviews/creation of interview questions for the study would have been nice. I'm really grasping at straws here cause the whole experience has been fantastic.* | *I can't think of anything. It was a fantastic experience working with this group of people.*  *Maybe just the beginning - I was a bit lost with all the quotes*  *I cannot think of anything we could have done differently.*  *Nothing for this project but if I had already done something like this project, maybe I would've liked being asked if I already had prior experience doing qualitative research so I could contribute more? Or asked to actively be involved in writing? I'm really just throwing stuff out there because it was a fantastic experience and I really wouldn't change a thing.*  *It would have been nice to have had a session where all patient partners [in the weekday group and weekend group] got to know one another.* |
