# Supplementary material for: Exploring Patient and Caregiver Perceptions of the Facilitators and Barriers to Patient Engagement in Research: Participatory Qualitative Study
Source: J Particip Med. 2025 Sep 30;17:e79538. doi: 10.2196/79538 (PMC12483476; doi:10.2196/79538)
Supplement: Multimedia Appendix 7 [file jopm-v17-e79538-s007.docx]

Multimedia Appendix 7: Select patient engagement resources identified by the patient-caregiver partners on our research team

| **1. Evaluation & Frameworks for Patient Engagement** | |
| --- | --- |
| **Resource** | **Description** |
| [Learning Together Evaluation Framework](https://ceppp.ca/en/resources/learning-together-evaluation-framework-for-patient-and-public-engagement-ppe-in-research/) | A comprehensive tool to evaluate the quality and impact of patient and public engagement in research. |
| [PEIR Plan Workbook](https://www.arthritisresearch.ca/wp-content/uploads/2018/06/PEIR-Plan-Guide.pdf) – Arthritis Research Canada | A workbook that helps researchers plan and evaluate meaningful patient engagement. |
| [Centre of Excellence on Partnership with Patients and the Public (CEPPP)](https://ceppp.ca/en/resources/) | Offers various tools and guides to foster effective partnerships with patients and the public in health research. |

| **2. Training & Education Modules** | |
| --- | --- |
| **Resource** | **Description** |
| [Research 101 (PORCCH)](https://porcch.ca/modules/) | Introductory training modules designed to build foundational knowledge in patient-oriented research. |
| [The BRICC Training – SCPOR](https://www.scpor.ca/upcoming-events/2021/7/29/building-research-relationships-with-indigenous-communities-training-module-online-event) | A module that supports respectful research relationships with Indigenous communities. |
| [Passerelle](https://passerelle-nte.ca/) | A digital learning platform offering training on patient engagement in research. |
| [RISE Learning Modules](https://my.riselms.ca/) | A platform offering courses and modules related to research impact and patient engagement. |
| [l'Unité de soutien SSA Québec (French training platform)](https://ssa.illuxi.com/?lang=french) | A French-language e-learning platform for training in patient-oriented research and systems transformation. |

| **3. National and Provincial SPOR SUPPORT Units** | |
| --- | --- |
| **Resource** | **Description** |
| [British Columbia SUPPORT Unit (BC SUPPORT Unit)](https://bcsupportunit.ca/) | Supports patient-oriented research across British Columbia through resources and partnerships. |
| [Alberta SPOR SUPPORT Unit (AbSPORU)](https://absporu.ca/patient-engagement) | Offers tools and guidance for patient engagement in research in Alberta. |
| [Saskatchewan Centre for Patient-Oriented Research (SCPOR)](https://www.scpor.ca/) | Provides support for patient-oriented research and training in Saskatchewan. |
| [Manitoba SPOR SUPPORT Unit (CHI)](https://www.chimb.ca/dhps/patient-engagement) | Leads patient engagement and health innovation in Manitoba. |
| [Ontario SPOR SUPPORT Unit (OSSU)](https://ossu.ca/for-researchers/patient-engagement-resources/) | A hub for patient engagement tools and resources in Ontario. |
| [Quebec SUPPORT Unit (Unité de soutien SSA Québec)](https://unitesoutiensrap.ca/) | Provides resources to promote patient-oriented research in Quebec. |
| [Maritime SPOR SUPPORT Unit (MSSU)](https://www.spor-maritime-srap.ca/) | Supports collaboration and patient engagement across the Maritime provinces. |
| [Newfoundland and Labrador SUPPORT Unit (NL SUPPORT)](https://nlsupport.ca/) | Promotes patient-oriented research and engagement in Newfoundland and Labrador. |
| [Yukon SUPPORT Unit (YSPOR)](https://yukonspor.ca/) | Encourages patient participation in research in the Yukon. |
| [Northwest Territories SUPPORT Unit (Hotıì ts'eeda)](https://nwtspor.ca/) | Promotes Indigenous knowledge and community-based patient engagement in the Northwest Territories. |
| [Nunavut SUPPORT Unit](https://www.nunavutspor.ca/) | A developing initiative to support patient-oriented research in Nunavut. |

| **4. National Organizations & Networks** | |
| --- | --- |
| **Resource** | **Description** |
| [Canadian Institutes of Health Research (CIHR) – Patient Engagement Resources](https://cihr-irsc.gc.ca/e/27297.html) | Provides a national framework and resources for meaningful patient engagement in health research. |
| [Healthcare Excellence Canada – Patient Engagement Hub](https://healthcareexcellence.ca/en/resources/patient-engagement-resource/) | A centralized hub offering best practices and guidance on patient partnerships. |
| [Research Impact Canada](https://rise.articulate.com/share/qV54-kftJACqH_QXUcaMODQ3W9qDw3-Y#/) | Offers training on maximizing the impact of research through engagement and knowledge mobilization. |
| [Neuromuscular Disease Network for Canada](https://neuromuscularnetwork.ca/cross-cutting-network-themes/expert-patient-capacity-building/patient-oriented-research-resources/) | Shares patient-oriented research tools specific to neuromuscular diseases. |

| **5. Patient and Family Engagement Programs** | |
| --- | --- |
| **Resource** | **Description** |
| [Canadian Cancer Survivor Network](https://survivornet.ca/) | Advocates for cancer survivors and offers engagement resources for research and healthcare. |
| [CanChild Family Engagement Program](https://www.canchild.ca/en/research-in-practice/family-engagement-program) | Focuses on promoting family engagement in child health research and services. |
| [McMaster University – Public & Patient Engagement Resources](https://ppe.mcmaster.ca/resources/) | Provides practical resources and guidelines for public and patient engagement. |
| [Child-Bright Network](https://www.child-bright.ca/ed-material) | Offers educational materials to support youth and families in patient-oriented research. |
