# Supplementary material for: Exploring Patient and Caregiver Perceptions of the Facilitators and Barriers to Patient Engagement in Research: Participatory Qualitative Study
Source: J Particip Med. 2025 Sep 30;17:e79538. doi: 10.2196/79538 (PMC12483476; doi:10.2196/79538)
Supplement: Multimedia Appendix 8 [file jopm-v17-e79538-s008.pdf]

CHECKLIST:
SELECT CONSIDERATIONS WHEN ENGAGING PATIENT/CAREGIVER PARTNERS IN RESEARCH STUDIES

1 Deciding to Engage Patient/Caregiver Partners

- ☐ **Researchers:** Seek out training and education from patient engagement organizations and mentorship from researchers with engagement experience.
- ☐ **Researchers:** Consider who you would like to engage and what perspectives/experiences they could bring to the study.
- ☐ **Researchers:** Set a clear but flexible vision for when, how, and why you want to engage patient/caregiver partners in your research.
- ☐ **Researchers:** Map out opportunities for patient/caregiver involvement across each stage of the research cycle.
- ☐ **Researchers:** Apply for funding or allocate existing funding towards patient/caregiver partner remuneration.
- ☐ **Researchers:** Designate a team member to facilitate engagement activities, provide personalized support to each patient/caregiver partner, and report back to the whole research team.
- ☐ **Researchers:** Consider how and when you will evaluate the quality of your patient/caregiver engagement activities (e.g., surveys like the PPEET and group discussions).
- ☐ **Researchers:** If applicable, gain institutional ethics board approval to engage patient/caregiver partners.

2 Recruiting Patient/Caregiver Partners

- ☐ **Researchers:** Create an intentional recruitment strategy that combines traditional methods (e.g., online recruitment) with tailored efforts (e.g., visiting community hubs).
- ☐ **Researchers:** Ensure recruitment materials are clearly written and culturally appropriate for intended audiences.
- ☐ **Researchers:** Learn more about prospective patient/caregiver partners by asking them questions in an interview or survey to ensure they are a good fit for the project.
- ☐ **Researchers:** If patient/caregiver partners must sign a consent form, ensure it is written in lay language and tailored to their role.

3 Orienting Patient/Caregiver Partners to the Study

- ☐ **Researchers:** Explain the purpose, aims, scope, and timeline of the research project to patient/caregiver partners in clear terms.
- ☐ **Researchers:** Explain the rationale behind the research to patient partners – are you informing policy, practice, or contributing to a body of literature?
- ☐ **Researchers:** Help patient/caregiver partners understand the context of the project by sharing background information/materials.
- ☐ **Researchers:** Present a menu of opportunities for involvement across each stage of the research cycle to patient/caregiver partners and clearly explain what involvement at each stage might entail.
- ☐ **Researchers:** Ask patient/caregiver partners if they have other skills or experiences that could contribute to the project.
- ☐ **Researchers:** Ensure patient/caregiver partners know which team member to contact about questions and concerns.
- ☐ **Researchers:** Present options for accessibility or training supports that you can offer to patient/caregiver partners.
- ☐ **Researchers:** Explain the availability of financial remuneration (i.e., compensation/reimbursement) to patient/caregiver parters and offer compensation alternatives if necessary.

- ☐ **Patient/caregiver partners:** Consider if the proposed engagement activities are a good fit for your strengths, interests, and current capacity.
- ☐ **Patient/caregiver partners:** Communicate with the research team if you need different accommodations/supports or if your accommodation needs change during the course of the project.
- ☐ **Researchers AND patient/caregiver partners:** Negotiate your roles and expectations together.
- ☐ **Researchers AND patient/caregiver partners:** Create a terms of Reference Document that outlines:
  - ☐ Names and roles of all team members.
  - ☐ Description of the mutually agreed-upon patient/caregiver partner role and activities.
  - ☐ Frequency and mode of communication.
  - ☐ Plans for compensation/reimbursement or compensation alternatives.
  - ☐ Plans for acknowledging patient/caregiver partner contributions (e.g., co-authorship).
  - ☐ Guidelines of conduct for all team members that promote safety, mutual respect, inclusiveness, co-building, and support.

4 Planning the Study Protocol

- ☐ **Researchers AND patient/caregiver partners:** Work together to plan the study protocol following the guidelines you established in your terms of reference.

5 Applying for Ethical Approval

- ☐ **Researchers:** Clearly explain to patient/caregiver partners why you must apply for ethical approval, what this process entails, and how long it usually takes.
- ☐ **Researchers:** Communicate regularly with patient/caregiver partners while awaiting ethics board review. Communication is essential even if there are no updates to share.
- ☐ **Researchers:** If the ethics review board requires changes to the study protocol, explain to patient/caregiver partners which aspects of the protocol must change and provide a rationale.

6 Conducting the Research

- ☐ **Researchers AND patient/caregiver partners:** work together to conduct the research following the guidelines you established in your Terms of Reference.
- ☐ **Researchers AND patient/caregiver partners:** Remember that your Terms of Reference is a living document – check in frequently to assess if the collaboration plans you made are working well.

- ☐ **Researchers:** Set aside time before or after each meeting for informal check-ins and relationship-building activities with patient/caregiver partners.
- ☐ **Researchers:** Provide education and training for patient/caregiver partners to support their engagement at each stage of the research cycle. Ensure that lay language is used in each meeting.
- ☐ **Researchers:** Show patient/caregiver partners the impact of their involvement by tracking changes in documents and engaging in multiple feedback cycles.
- ☐ **Researchers:** Consider power dynamics between researcher team members and patient/caregiver partners. Schedule a combination of "patient/caregiver partner only" and full team meetings.
- ☐ **Researchers:** Evaluate the quality of your patient/caregiver engagement at the mid-point and end of your research project to identify and address challenges.

7 Knowledge Translation

- ☐ **Researchers:** Initiate a conversation and set clear expectations about co-authorship with patient/caregiver partners. Do not list a patient/caregiver partner as a co-author on knowledge-translation materials if you have not asked them first.
- ☐ **Researchers:** Contribute to a community of practice by reporting your engagement activities and evaluations of patient/caregiver engagement using guidelines such as the GRIPP2 checklist.

- ☐ **Researchers AND patient/caregiver partners:** Work together to identify target audiences for the research findings.
- ☐ **Researchers AND patient/caregiver partners:** Decide which knowledge translation materials you will work together on (e.g., manuscripts, posters, videos, infographics).
- ☐ **Patient/caregiver partners:** Know that you have choices around how your name appears in an author list. You can use your full name or a pseudonym if you prefer not to be directly identified.
